# Comparison of multiplex meta analysis techniques for understanding the acute rejection of solid organ transplants

**DOI:** 10.1186/1471-2105-11-S9-S6

**Published:** 2010-10-28

**Authors:** Alexander A Morgan, Purvesh Khatri, Richard Hayden Jones, Minnie M Sarwal, Atul J Butte

**Affiliations:** 1 Department of Pediatrics, Stanford University School of Medicine, Stanford, CA 94305, USA; 2 Center for Biomedical Informatics Research, Stanford University School of Medicine, Stanford, CA 94305, USA; 3 Lucile Packard Children’s Hospital, 725 Welch Road, Palo Alto, CA 94304, USA

## Abstract

**Background:**

Combining the results of studies using highly parallelized measurements of gene expression such as microarrays and RNAseq offer unique challenges in meta analysis. Motivated by a need for a deeper understanding of organ transplant rejection, we combine the data from five separate studies to compare acute rejection versus stability after solid organ transplantation, and use this data to examine approaches to multiplex meta analysis.

**Results:**

We demonstrate that a commonly used parametric effect size estimate approach and a commonly used non-parametric method give very different results in prioritizing genes. The parametric method providing a meta effect estimate was superior at ranking genes based on our gold-standard of identifying immune response genes in the transplant rejection datasets.

**Conclusion:**

Different methods of multiplex analysis can give substantially different results. The method which is best for any given application will likely depend on the particular domain, and it remains for future work to see if any one method is consistently better at identifying important biological signal across gene expression experiments.

## Background

Messenger RNA expression measurement with microarrays enables a very highly multiplexed examination of the relative expression of genes under different experimental conditions, and several different techniques for combining the results of different microarray experiments have been proposed [1-5]. However, there has been very little work done looking at the differences between meta analysis techniques [6] and how they may be used together to understand a concrete biomedical problem with clinical implications. By looking at a particular biomedical problem, in this case acute rejection of solid organ transplants, we can compare the results of different meta analysis approaches, both to one another and what is known about the process of transplant rejection.

In 2007, more than 28,000 solid organs (including heart, liver, lung, pancreas, kidney and intestine) were transplanted in the United States [7]. Unfortunately, recipients of allografts are subject to the problems of transplant rejection. Particularly dire is the activation of a significant T-cell mediated response against the implanted tissue, termed acute rejection. Therefore, we can expect that samples taken from patients undergoing acute rejection will have substantially increased expression of genes associated with a T-cell mediated immune response than samples taken from patients not undergoing acute rejection. However, the mechanism by which T-cells infiltrate the allografts and mediate rejection is not known. It can be hypothesized that although the prompts for tissue-specific injury may be different, there may be common mechanism of rejection (e.g., T-cell infiltration) across all solid organ transplants. Identification of shared mechanisms that lead to tissue-specific destruction can facilitate novel treatments without requiring the understanding of individual, tissue-specific mechanisms. However, the conventional experiment design, where only one type of solid organ allograft is examined in an experiment is inadequate to identify such a mechanism. In this regard, microarray data from different solid organ transplants, available from public data repositories such as NCBI Gene Expression Omnibus (GEO), can be combined to gain a greater understanding of the process of acute rejection to aid in early diagnosis and the tailoring of treatment regimens.

However, these experiments are performed on different microarray platforms by different laboratories, Several techniques for combining the results of different microarray experiments have been proposed and the theoretical differences between methods have been presented and a range of methods have been covered in review articles [1-5]. However, there has been very little work done looking at the empirical differences between meta analysis techniques. Furthermore, most previous work has used simulated data or measures of consistency [6]. We are most interested in how meta-techniques may be used together with real data to understand a concrete biomedical problem with clinical implications.

Multiplexed meta analysis differs from traditional, single measurement, approaches in the number of variables being simultaneously combined across experiments. In traditional meta analysis, a given variable is combined across several experiments which may be measuring the exactly the same feature or slightly different features that will be combined, and there is an extensive set of techniques for this form of analysis [8]. However, in multiplex meta analysis, many variables are being simultaneously combined across experiments. Some of the experiments may not have measured that particular variable, and some of the experiments may have measured that variable multiple times within a single experiment (such as multiple probes measuring expression of the same gene on a microarray).

In single measurement approaches, it is often important to identify whether or not the experiments combined are comparable in design and consistent in result. However, in multiplexed meta analysis, we are often trying to identify which variables represent a core of similar behavior across experiments and it is not essential that the other variables are homogeneous in variation.

Finally, multiplexed meta analysis is often used for data-mining purposes to identify particularly strongly associated variables across experiments, such as the development of biomarkers and other forms of hypothesis generation that will be validated experimentally. It is therefore often acceptable to sacrifice significant amounts of sensitivity for a high degree of specificity when analyzing tens of thousands of variables.

In this work, we focus on the application of some of the traditional approaches of meta analysis applied to multiplex gene expression data. We show that for the very basic task of developing a prioritized gene list, our applied parametric approach with known flaws actually outperforms non-parametric methods that should have greater accuracy at the level of any individual gene. For multiplex problems, the large error bars associated with the estimation of meta fold-change for any particular gene is less important than the overall average improvement in gene ranking that this meta analysis method provides.

## Results

Traditional meta analysis methods may be grouped into two broad categories of approach. The first is to use a non-parametric method to combine the significance results of the different experiments being amalgamated in the meta analysis. At the simplest level this can include counting methods that look at the proportion of experiments showing significance. More accurate tests include omnibus methods that combine p-values to obtain a meta p-value. The second general approach is to try to merge a series of predictions from separate experiments to develop a model of effect size. For gene expression this would amount to a meta fold-change estimate. We examined the most commonly used examples of both approaches.

There is a range of non-parametric omnibus methods for combining p-values from different studies. The basic assumption of most methods is that in the absence of any actual difference in a variable being measured, the p-values should be uniformly distributed. We will focus on one of the best studied, Fisher's method, which combines the squares of the p-values, [*p_i_*], from each of the *k* studies and compares that to a χ^2^ distribution:

The resulting list of meta p-values, one for each gene, can then be subject to correction for multiple hypothesis testing or used to estimate a false discovery rate and obtain a q-value [9].

As an underlying statistical test, we used two one sided t-tests, one for a hypothesis of increased expression, another for a hypothesis of decreased expression. Because Fisher's method does not take into account the sign of the variation, we examine the hypothesis of increased expression separately from decreased expression, and then for each gene, take the minimum p-value. Taking the minimum of two values introduces a very slight bias, but our primary interest is in creating a ranked list. For the combination of these five microarray studies, we get a substantially enriched selection of differentially expressed genes over random; 5.2% of the 20,113 genes studied have a meta p-value less than 0.01 for the hypothesis of increased expression (1% expected by chance), whereas 6.5% have p-values less than 0.01 for the hypothesis of decreased expression (1% also expected by chance). When the minimum p-value of the Fisher's method applied to the two one-sided t-tests was corrected for multiple hypothesis testing through the method proposed by Benjamini and Hochberg [10], 584 genes had a corrected p-value less than 0.01; this suggests a substantial enrichment for true positives.

Another important purpose of meta analysis is to model the magnitude, i.e. the effect size, of the variation. For gene expression measurements, this corresponds to combining fold-changes across studies to identify a meta-fold-change that is an amalgamation of the constituent studies. One commonly used method is to take a linear combination of effect sizes (fold-changes in this case, *f_i_*), weighted by the variance in the effect size within each study (*w_i_*), with the confidence intervals combined with the same weights. This means that studies with larger intra-study variation (noise) contribute less to the overall estimate of fold-change.

Unfortunately, this approach makes a strong assumption about the distribution of the data and how it may be combined. For multiplexed meta analysis, there are many variables each with possibly very different distributions, and it is very difficult, if not impossible, to identify any single best distribution to model the data, and there are generally insufficient samples to develop separate models for each variable (gene). That means that this is neither a robust nor a powerful statistical test for each variable.

The non-parametric methods of p-value combination make no assumptions about the underlying distribution of the data, and rely only on the results of the underlying statistical methods used to analyze that data. Methods such as the t-test are very robust and accurate when looking at gene expression measurements. Indeed, they are more accurate at detecting differential expression than fold-change [11]. However, the fold-change is still an important measure of effect size, and it is substantially more reproducible across studies [12]. For instance, if gene expression studies are being combined to suggest proteins for further analysis, a very small gene expression fold-change is not likely to lead to a measurable difference in protein expression, independent of the statistical significance of the difference.

The difference between significance and effect size is an important distinction in applied statistics in general. Although, statistical significance is often associated with large effect size, they are not equivalent as demonstrated in the commonly used 'volcano plot' to display gene expression data [13]. For another example, a correlation coefficient is a measure of effect size, whereas a p-value on a measure of correlation is a significance measure. When doing most types of analysis, we want both a significance estimate and an effect size estimate. In multiplexed meta analysis, we suggest combining a robust measure of significance (such as a t-test or modified t-test) using one of the non-parametric omnibus methods to identify significant difference with a parametric method of combining effect size estimates to get a reasonable estimate of effect size (i.e. fold-change).

The meta p-values obtained by Fisher's method and the meta-fold-change value obtained by the invariance method can be plotted for each gene, leading to a meta analysis version of the volcano plot [14]. A typical volcano plot graphs p-value against fold-change and shows that extremely high and extremely low fold-changes tend to be associated with highly significance p-values, and has a marked triangle shape.

**Figure 1:**
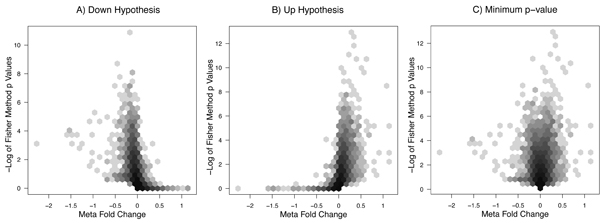
Meta fold-change obtained from the inverse variance method plotted against -log_10_(p_meta, Fisher_) for each gene, counts smoothed with hexbin [27]. Panel A shows the Fisher method meta p-values obtained from a one sided t-test checking against lowered expression of the gene. Panel B shows the Fisher method applied to p-values from a one sided test against a hypothesis of increased expressed. The third panel, C, shows the minimum of the two Fisher method obtained values obtained for each gene plotted against the meta fold-change. Note that although a meta p-value for a lowered expression is indeed associated with a lower meta-fold change, and vice versa, overall extreme values in meta fold-change are not associated with extremely significant p-values. Panel C which combines the two tests does not show the extreme, marked forked structure that is characteristic of a "volcano plot". One of the key features of a nonparametric approach like Fisher's method is that it allows small changes in fold change that are consistent across experiments to rise to statistical significance across studies. It is important to note however, that these meta analysis results show that meta fold-change and meta p-value can be more decoupled than they are in a single study, and that the two methods can give different rank prioritizations of genes that differentially expressed across studies.

Interestingly, we see in Figure 1 that the meta analysis results are not separated into the forked distribution of a traditional volcano plot. It shows the characteristic triangular shape only slightly, if at all. This means that genes that are highly significant in differential expression across the experiments (significant meta p-value) can have a very small meta-fold-change estimate, and the reverse. We see that these two methods do not have a clear relationship with each other. The coefficient of correlation between the log significance values and the absolute value of the meta fold-change is 0.3, which is not nearly as dramatic as might be expected. It is certainly possible that genes that differ in expression magnitude (meta fold-change) only modestly may show a significant difference across experiments as captured by Fisher's, non-parametric, method. This is indeed what may be seen in Figure 1. This suggests that Fisher's method might be superior at identifying some consistently differentially expressed, low magnitude, signal that is biologically important. However, for our chosen evaluation, this does not seem to be the case.

**Figure 2:**
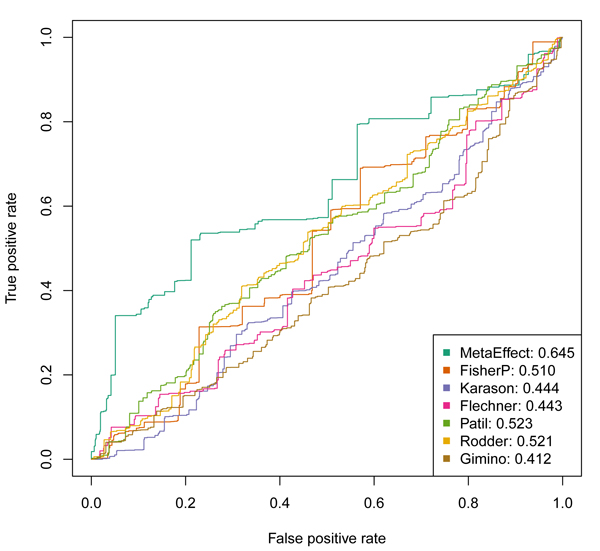
Receiver Operating Characteristics (ROC curves) showing the relative ability of the meta fold-changes and the meta p-values to prioritize genes associated with "IMMUNE RESPONSE" as identified in the Molecular Signatures Database. The ability of individual studies to identify/prioritize genes is also shown, indicated by the last name of the first author of the associated publication. In the figure legends, the different sources of the ROC curves are indicated by different colors and the area under the curve is shown to the right of the label. The greater the area under the curve, the great predictive power to identify genes involved in immune response. Note that the meta fold-change (indicated by MetaEffect for the meta effect estimate approach) has the greatest predictive power. The Fisher's method (FisherP) meta p-values do better than most of the single experiments, but are completely dominated by the meta fold-change.

**Figure 3:**
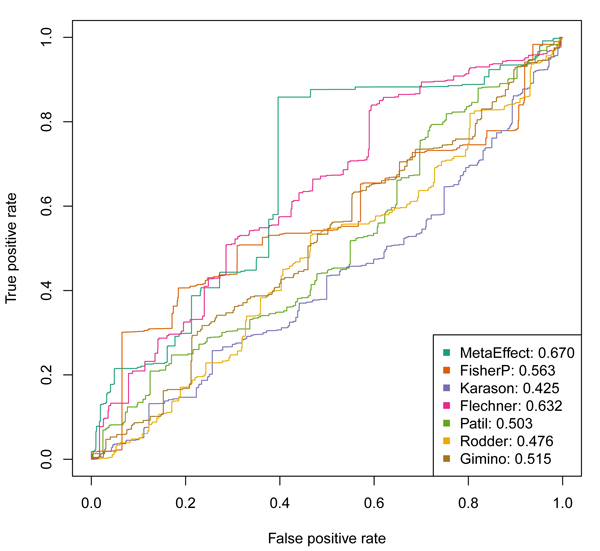
ROC curves showing the relative ability of the meta fold-changes and the meta p-values to prioritize genes associated with "DEFENSE RESPONSE" as identified in the Molecular Signatures Database. Note that the overall area under the curve is superior for the prioritization of genes via meta fold-change from the inverse variance meta effect size estimate (MetaEffect), and this method is better at identifying genes at the very top of the list, as indicated by the dominance of the ROC curve at the far left, indicating very strong enrichment (higher true positive rate), while the number of false positives are still relatively low. This result suggests that the meta fold-changes provide a superior method for identifying the key genes in the undesirable immune response in acute organ rejection.

To attempt to identify which method is better at identifying biologically relevant genes in this data set, we use the Molecular Signatures Database [10] to identify the lists of genes associated with "Immune Response" and "Defense Response". A gene expression meta analysis approach that is being used to probe the undesirable immune response in transplant rejection should prioritize these genes more highly. We can compare the prioritization provided by Fisher's method p-values (using the lower p-values as indicating higher priority) and the meta fold-change provided by the inverse variance method to select these import immune function genes. These results are shown in Figures 2 and 3. It can be seen that the ranking provided by the effects size estimate from the inverse variance method, the meta fold-change, is superior to that provided by Fisher's method, the meta p-values. The Fisher's method also does not differentiate itself from the prioritization provided by the individual constituent datasets being combined.

## Discussion

It is important to evaluate the ability of different approaches to multiplex meta analysis to answer the questions being asked. Unfortunately, just as there is no single question asked of gene expression measurements, there is no one correct way to evaluate different gene expression analysis techniques, even with extensive biological validation.

In this pilot application, we were interested in obtaining a prioritized list of the genes involved in acute rejection across the types of transplanted organs. We have a strong prior expectation that this common process of acute rejection involves an immune response reaction. We searched for genes known to be involved in immune response (annotated as “immune response” genes), as reported by the Molecular Signatures Database [15], and observe how the different meta analysis approaches prioritize those genes. Our method of evaluation here was to count the number of immune related genes at each point of depth into the ranked list.

We find the meta fold-change derived by the inverse variance method is better at recapitulating our prior expectation of immune response genes than any single dataset, at every depth into the gene list (Figure 2). Interestingly, the list of genes obtained by ranking according to the meta p-values obtained by Fisher's method are no better than the best individual experimental results at identifying and prioritizing the genes of interest. Though we acknowledge this is only a preliminary analysis, these results suggest that the ordering of genes provided by meta effect size estimate may be more biologically relevant, even if non-parametric methods of significance may be more accurate for any individual gene.

## Conclusions

Our results show that there is value to be gained in doing meta analysis and combining results from different studies can improve the ranking of genes. However, not all meta analysis techniques are equivalent, and not all seem to provide the same level of improvement over looking at individual experiments.

Effective, useful multiplex analysis techniques for gene expression analysis must be able to provide accurate estimates of statistical significance of differential expression. However, we will continue to need general, overall estimates of effect size (fold-change). Indeed, our results suggest that for at least some biological questions these estimates of fold-change are more useful for identifying features important to the biology of the problem than meta significance estimates (p-values).

The study of gene expression measurements in acute rejection offers a particularly good opportunity to investigate methods of multiplex meta analysis. We know that a powerful immune response is involved, and the fact that the meta fold-change approach provides a greater enrichment of these genes implies that it is capturing more of the core, shared process of acute rejection across organ types. At the same time that we generally know that acute rejection involves an immune response, we are interested in the particular genes and pathways involved and can investigate the highly ranked genes more thoroughly and look for biological validation to help address the important clinical problem of organ rejection.

In this comparison of two approaches to multiplex meta analysis, we have identified some of key issues that need to be investigated further. The fact that two of the most common approaches to traditional meta analysis, a nonparametric significance test based approach and a parametric, effect size estimate approach, give very different results highlights many of the challenges. This is an opportunity for future efforts, as we can investigate methods that combine both of these approaches. It will also be important to identify in future work if one of these two methods or another approach is able to consistently identify important biologically relevant signals across gene expression experiments. The evaluation we have chosen, the prioritization of immune relevant genes in transplant rejection expression variation datasets is far from a perfect evaluation metric. Future comparisons will need to look at other biomedically important problems and gold-standards to identify if any one approach of family approaches can be shown to provide better results than another.

## Materials and methods

We chose to search for a common signature of organ transplant rejection as a test of our methods. We collected data from five publicly available gene expression studies (Table 1) on transplant rejection using Affymetrix single color arrays: two on kidney [16,17], two on lung [18,19], and one on heart [20].

**Table 1:** Overview of the datasets.

Dataset	Organ	AR	Tol	Platform	GEO Acc.
Gimino, et al.	Lung	7	27	hg133a	GDS999
Karason, et al.	Heart	3	6	hg133a	GDS1684
Patil, et al.	Lung	18	14	hg133a	GSE6095
Flechner, et al.	Kidney	7	10	hgu95a	GDS724
Rödder, et al.	Kidney	18	8	hg133plu2	GSE9493

**AR** indicates number of tissue samples of acute rejection, **Tol** indicates the number of samples of tissue from graft tolerant patients.

The individual data sets were taken from the Gene Expression Omnibus [21] and the data was quantile-quantile normalized and analyzed using Bioconductor [22]. Two one sided t-tests were performed for each probeset within each of the five constituent datasets. Fisher's method was then performed as previously described [8] by pooling all the p-values for the probesets for each gene. Probes were mapped to Entrez Gene identifiers [23] using AILUN [24]. The minimum of the two p-values (one from each of the two one-sided tests which were synthesized using Fisher's method) for each gene was taken as the meta p-value. Within each experiment, a fold change was calculated for each gene by taking the log ratio of the geometric means of the expression values between the samples showing acute rejection and those showing stable acceptance of the transplanted organ. The geometric mean of expression value is equivalent to taking the arithmetic means of the log expression values. The sample variance was also calculated for this dimensionless quantity derived from fold change (a ratio), and then the fold changes were combined for each gene using the previously described linear model, weighted by inverse variance, to provide an effect size estimate, in this case a meta fold-change. A standard deviation on this effect size estimate was calculated using a fixed effects model [8].

Gene signatures for "Immune Response" and "Defense Response" were taken from the Molecular Signatures Database [15], and mapped to our genes through their gene symbols. These signatures were compared to the rankings provided by the two meta analysis methods, and ROC curves were produced using the ROCR package [25]. The multtest package [26] was used for correction of the p-values for multiple hypothesis testing.

## Competing interests

The authors declare that they have no competing interests.

## Authors' contributions

RHJ and PK collected annotated the expression datasets. AAM designed the experiments, performed the analysis and wrote the paper draft. PK and AAM worked on the interpretation of results. PK, MMS and AJB helped in planning, discussing, and writing the paper.
